# Giant retinal tear after intra-arterial chemotherapy for advanced unilateral retinoblastoma

**DOI:** 10.1186/s40942-017-0083-x

**Published:** 2017-08-14

**Authors:** Camila V. Ventura, Audina M. Berrocal, Jennifer Thomson, Fiona J. Ehlies, Azeema Latiff, Timothy G. Murray

**Affiliations:** 1 Department of Ophthalmology, Bascom Palmer Eye Institute, University of Miami Miller School of Medicine, 900 NW 17th Avenue, Suite 262, Miami, FL 33136 USA; 2 Murray Ocular Oncology and Retina, Miami, FL USA

**Keywords:** Retinoblastoma, Intra-arterial chemotherapy, Rhegmatogenous detachment

## Abstract

**Background:**

Retinoblastoma is considered the most common intraocular malignancy in childhood, comprising 4% of all pediatric cancers. Management of retinoblastoma has evolved over the past two decades and intra-ophthalmic artery chemotherapy has emerged as a new modality of globe-conserving treatment with excellent results. This treatment achieves effective tumor reduction by delivering localized chemotherapy, decreases enucleation rate, and minimizes systemic and local side effects.

**Case presentation:**

We report the case of an 8-year-old girl with a late presentation of an advanced unilateral retinoblastoma associated to diffuse exudative retinal detachment in the right eye, classified as group E by the International Classification of Retinoblastoma. The initial therapeutic proposal for the patient was five sessions of intra-ophthalmic artery chemotherapy (IAC) associated to large spot diode laser therapy. After undergoing four sessions of IAC, the fundus exam revealed a giant retinal tear associated to a total retinal detachment in the affected eye. The IAC treatment was concluded and enucleation was considered the best treatment option at that moment, since IAC was unable to control the tumor’s activity and the patient’s eye presented with a complex rhegmatogenous retinal detachment (RRD). However, family left for a second opinion and never returned.

**Conclusions:**

The usage of IAC for retinoblastoma management may lead to important local complications. Despite rare, RRD secondary to IAC may occur. We postulate that the giant tear observed in this case was caused by the rapid tumor necrosis using this route of treatment.

## Background

Retinoblastoma (RB), a neuroectodermal tumor originated from the inner layer of the optic cup, was first described as a specific entity by James Wardrop in 1809 [1]. It is considered the most common intraocular malignancy in childhood, comprising 4% of all pediatric cancers, with about 200–300 new cases every year in the United States (US) [1]. RB tumors have no sex predilection and can be either heritable, which is associated with a germline mutation of RB1 gene, or non-heritable [2]. Heritable mutations typically present in the 1st year of life with bilateral disease. Non-heritable form, in the other hand, presents slightly later in life and is primarily unilateral [2]. Although RB may present at any age, its’ appearance after 5 years of age is considered rare [3].

The survival rate of retinoblastoma is still low, with rates ranging from 50 to 70%. However, in developed countries the survival rate has improved to almost 100%, and so have the visual outcomes [2, 3]. This improvement can be associated to the early recognition and advances in management. Since the tumor presents with a remarkable chemotherapy-sensitivity, there has been a shift for treating RB patients from radiotherapy to chemotherapy [2–4].

Nowadays, there are various routes of administration of chemotherapy treatment including intravenous, intra-arterial, periocular, and intravitreal. The choice for a route of administration depends upon the tumor laterality and tumor staging [4]. Chemotherapy agents delivered directly into the ophthalmic artery (intra-ophthalmic artery chemotherapy; IAC) was first explored in the 1950s by Reese et al. with intracarotid administration of triethylene melamine in RB patients [5]. In 1999, Kaneko et al. described the selective ophthalmic arterial injection of melphalan by distal occlusion of internal carotid artery with a balloon catheter [6]. This technique was further modified by Abramson et al. who described the supraselective injection of the drug directly into the proximal portion of the ophthalmic artery [7].

The systemic side effects caused by IAC are not significant since it offers minimum systemic absorption of drugs. However, its protection against systemic metastasis, pinealoblastoma, and secondary cancers remains a controversy of this treatment [4]. Severe local complications include vitreous and/or retinal hemorrhage, retinal detachment (RD), occlusive vasculopathy, retinal pigment epithelial changes, choroidal and retinal ischemia, chorioretinal atrophy, and phthisis bulbi [1, 3, 4, 8–11].

The most common types of RD associated to RB are tractional and rhegmatogenous. Generally, rhegmatogenous retinal detachments (RRD) are caused by breaks adjacent to cryotherapy scars or ablative therapies applied locally [9, 10, 12]. However, RRD may also occur as a direct complication of IAC. This specific complication is usually related to the rapid full-thickness tumor regression leaving atrophic retinal hole [13]. Although considered rare, herein, we describe a RB case that presented with a giant retinal tear followed by RRD after IAC.

## Case presentation

An 8-year-old girl was urgently referred for leukocoria. The patient reported central vision loss and floaters in her right eye for a month. Her family history and past medical history were noncontributory. At presentation, her best-corrected visual acuity was 20/250 and 20/20 in the right and left eye, respectively. The diagnosis of retinoblastoma (RB) was confirmed after slit-lamp, fundus, and ultrasound examination and classified as group E by the International Classification of Retinoblastoma (ICRB). She was taken to the operating room after being submitted to brain and orbital MRI that revealed no central nervous system involvement.

**Fig. 1:**
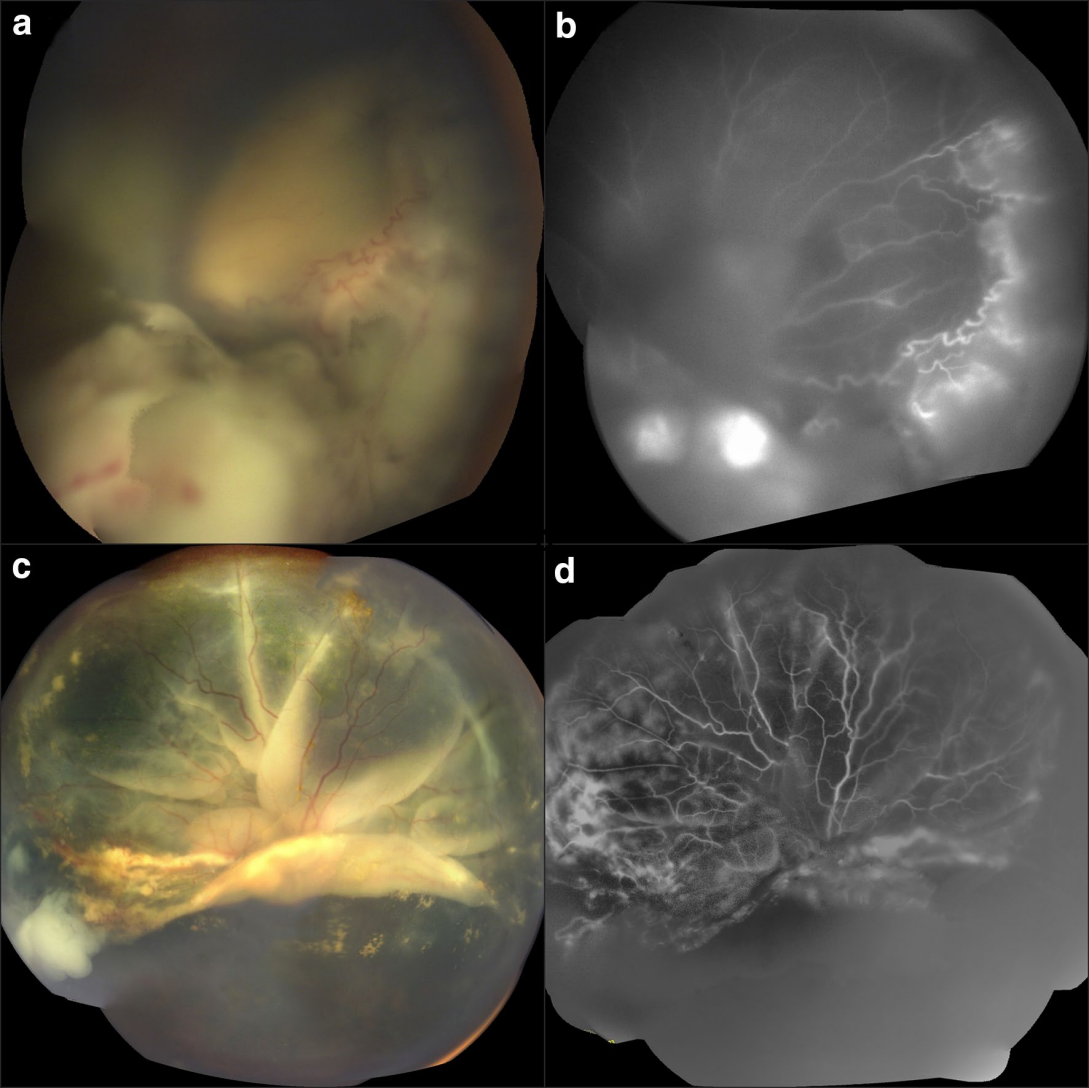
Fundus and fluorescein angiography images of the patient’s right eye prior to treatment (**a**, **b**) and post four cycles of intra-arterial chemotherapy (**c**, **d**). **a** Fundus image showing a multi lobulated retinoblastoma with vitreous seeding and extensive exudative retinal detachment; **b** fluorescein angiography image showing gross hyperfluorescence in the inferior and nasal quadrants; **c** fundus image showing a giant inferior retinal tear and tumor activity in the temporal periphery; **d** fluorescein angiography image showing a giant inferior retinal tear and diffuse retinal hyperfluorescence

In the operating room, a dense multilobulated advanced RB with an extensive exudative retinal detachment (ERD) and vitreous seeding was observed (Fig. 1a, b). The left eye showed no involvement. The treatment plan established for the patient was five cycles of IAC with three drugs (30 mg of carboplatin, 0.4 mg of topotecan, and 7.5 mg of melphalan) supplemented with large spot diode laser ablative therapy. The patient underwent surgical management where the balloon occlusion technique was used as a vascular access, without any complication.

After completing four cycles of IAC treatment with adjacent transpupillary thermal large spot size diode laser ablation, a massive reduction of the tumor’s volume was noticed; however, the ERD persisted. Three weeks later, an inferior giant retinal tear was observed in the right eye (Fig. 1c, d). After completing the 5th cycle of IAC, the patient was submitted to a thorough ophthalmological examination that evidenced extensive tumor activity and a complex RRD. The decision then was to perform an enucleation of the affected eye. However, family opted to get a second opinion and never returned.

## Discussion and conclusion

With therapeutic advancement, IAC has become the preferred route of chemotherapy administration for unilateral RB cases [13]. However, despite providing a more powerful tumor control and greater salvage of advanced RB compared intravenous chemotherapy, local complications secondary to IAC treatment may occur.

Previous studies have reported tractional and RRD as possible complications of IAC treatment. Most of these retinal breaks associated with RB treatment are caused by the local ablative therapy that mechanically causes the retinal holes that leads to a RD [8, 10, 12]. However, the particularity of the current case is the different pathogenesis of the tear as well as its extension. We believe that in this specific case, the rhegmatogenous component of the retinal detachment was caused by tractional forces in response to the tumor’s volume reduction during IAC treatment. To corroborate with this hypothesis, Shields et al. [13] recently reported RRD cases in patients submitted to IAC with no adjacent local consolidation therapy such as cryotherapy, transpupillary thermotherapy, or argon laser photocoagulation. Similarly, they postulated that their cases were related to the rapid tumor regression that caused atrophic retinal holes or horseshoe tears.

Despite the slightly increase of post-treatment RRD using IAC, this complication is still considered rare with an estimated incidence of 6% [13]. According to Shields et al., these RRD cases are most likely to occur in eyes with advanced RB (Group D or E), which was also noticed in our case report [13].

It is important to highlight that none of the eyes reported by Shields et al. presented with such an extensive retinal tear. Managing the RRD in eyes with RB tumors after being treated primarily with IAC can be quite challenging. The first concern is to maintain complete tumor control before repairing the RRD and consequently, analyze its potential to be repaired [13]. Since this was not the case of our patient, the decision was to enucleate the affected eye. However, the family wanted to seek for a second opinion and never returned for a follow-up.

In conclusion, IAC can provoke retinal tears as tractional forces are created during the tumor’s contraction, precluding globe salvage. Thus, RB patients undergoing IAC treatment should be closely monitored for early detection of complications and better management of the disease.

## Abbreviations

RB: retinoblastoma
US: United States
IAC: intra-arterial chemotherapy
RD: retinal detachment
RRD: rhegmatogenous retinal detachments
ICRB: International Classification of Retinoblastoma
MRI: magnetic resonance imaging
ERD: exudative retinal detachment
